# Acetabular Fractures with Central Hip Dislocation: A Retrospective Consecutive 50 Case Series Study Based on AO/OTA 2018 Classification in Midterm Follow-Up

**DOI:** 10.1155/2021/6659640

**Published:** 2021-09-17

**Authors:** Chun-Yen Chen, Chin-Jung Hsu, Tsung-Li Lin, Hsien-Te Chen, Chun-Hao Tsai

**Affiliations:** ^1^Department of Orthopedics, China Medical University Hospital, Taichung, Taiwan; ^2^School of Chinese Medicine, China Medical University, Taichung, Taiwan; ^3^Department of Sports Medicine, China Medical University, Taichung, Taiwan; ^4^Graduate Institute of Biomedical Sciences, China Medical University, Taichung, Taiwan; ^5^Spine Center, China Medical University Hospital, China Medical University, Taichung, Taiwan

## Abstract

**Introduction:**

Management of acetabular fractures is challenging, especially when a medial acetabular fracture is complicated by central hip dislocation. We retrospectively investigated the clinical outcome and risk factors of secondary hip osteoarthritis requiring total hip arthroplasty after the surgical treatment of acetabular fractures with central hip dislocation.

**Materials and Methods:**

The medical records of all patients who had acetabular medial wall fractures with central hip dislocation treated with open reduction and internal fixation by a single surgeon between January 2015 and June 2017 were reviewed. Surgical reduction was performed with the modified Stoppa with/without the Kocher-Langenbeck (KL) approach. Patients were followed for a minimum of three years, and the Majeed scoring system was used for functional evaluation. Multivariate logistic regression analysis was used to assess the association of patients' characteristics with the likelihood of advanced posttraumatic arthritis developing with conversion to total hip arthroplasty.

**Results:**

Fifty patients were included in this study, with disease classified as AO/OTA 2018 62B/62C. Thirty-five patients (70%) had good or excellent Majeed pelvic scores. Eleven patients (22%) eventually received total hip arthroplasty because of end-stage posttraumatic arthritis. Three risk factors identified for total hip arthroplasty were male sex, initial marginal impaction, and sciatic nerve injury. Kaplan-Meier survivorship analysis estimated that the cumulative probability of free-from-end-stage arthritis was 78% (95% confidence interval, 73%–90%) at the 5-year follow-up.

**Conclusion:**

Surgical fixation with the modified Stoppa and the KL approach for acetabular medial wall fractures with central hip dislocation is an effective approach with a satisfactory functional outcome. A prodromal factor was marginal impaction concomitant with articular damage. The trauma of high axial loading and the occupational distribution (males performing heavy manual labor and heavy lifting) with preoperative sciatic nerve injury increased the odds of developing end-stage arthritis.

## 1. Introduction

The reported worldwide annual incidence of acetabular fractures is about 3 per 10000 persons [1]. More than 60% of acetabular fractures are associated with high-energy injuries, especially high-speed motor vehicle accidents [2]. However, cases of acetabular fractures with hip dislocation are rare [3].

Hip dislocation can be divided into anterior, posterior, and central dislocations. Acetabular fractures with central hip dislocation result from direct axial loading through the greater trochanter in abduction [4, 5]. These fractures account for 20% of anterior fracture patterns and 33% of T-shaped fractures of the acetabulum [6]. Moreover, the intrapelvic displacement of the femoral head may cause comminuted fractures of the anterior column and quadrilateral plate [7]. In patients with osteoporosis, impaction injuries to the cartilage [6] and concomitant femoral neck or intertrochanteric femur fractures can occur.

Only a few studies have investigated the treatment and prognosis of traumatic acetabular fractures with central hip dislocation, and the incidence and predictors of secondary hip osteoarthritis after central hip dislocation are unclear. Westerborn [8] first described, in 1954, the concept of central dislocation of the femoral head and treated it with arthroplasty. A case series with a long-term review reported damage to the femoral head as a predictor of posttraumatic arthrosis [9]. Recent case reports have illustrated the quadrilateral plate fracture with central hip dislocation [10–13]. However, the treatment and outcome of these cases varied. A systematic review reported various outcomes and treatment modalities for acetabular fractures with central hip dislocation, including conservative methods such as skeletal traction, close reduction with percutaneous screws, open reduction with plating, and iliofemoral external distraction, and one-stage total hip arthroplasty (THA) [3, 14]. However, comprehensive classification of acetabular fractures with central hip dislocation remains controversial [15]. Consensus about the surgical strategies, outcomes, and risk factors of secondary hip osteoarthritis is lacking.

In the present study, we reclassified our cohort of central hip dislocation according to the 2018 Arbeitsgemeinschaft für Osteosynthesefragen/Orthopaedic Trauma (AO/OTA) Fracture and Dislocation Classification Compendium [16]. This study evaluated the clinical outcomes and risk factors of secondary hip osteoarthritis after surgical treatment of acetabular fractures with central hip dislocation. We hypothesized that certain fracture patterns, associated injuries, perioperative factors, and patient factors predispose patients to postoperative hip osteoarthritis.

## 2. Materials and Methods

### 2.1. Study Design and Population

**Figure 1:**
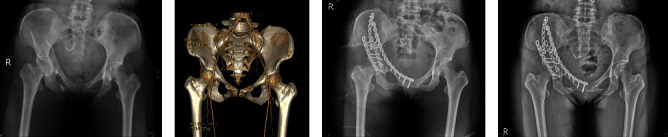
(a) Radiograph of a 47-year-old woman with acetabular fractures and central hip dislocation (AO/OTA 62B3.2). (b) Three-dimensional CT reconstruction. (c) Postoperative radiograph of using dual approaches. (d) Radiograph at final follow-up 405 days after surgery.

This nonrandomized retrospective cohort study was conducted between January 2015 and June 2017. All patients with traumatic acetabular fractures and central hip dislocation had received an open reduction and internal fixation with locking plates. The inclusion criteria were the presence of closed acetabular fractures with central hip dislocation and age of >20 years. Patients with fractures involving the bilateral pelvis ring, open fractures, multiple fractures, or a follow-up period of <3years were excluded. Surgery in all cases was performed by a single surgeon who specialized in orthopedic traumatology. A preoperative radiographic survey with plain radiographs and computed tomography (CT) scans of the pelvis with three-dimensional reconstruction was conducted for fracture characterization. At operation, the patients were placed in a lateral decubitus position. Based on the fracture patterns, the Kocher-Langenbeck (KL) approach and modified Stoppa approach were applied. In patients with AO/OTA 62B transverse-type fractures but no posterior column involvement, only the modified Stoppa approach was used. Dual approaches were employed to access the posterior column or posterior wall (Figure 1). We performed autologous bone grafting from the iliac crest through the lateral window in the modified Stoppa approach and posterior wall fracture in the KL approach in case with marginal impaction. Postoperative anteroposterior and Judet views of the pelvis were obtained. Using the Matta reduction criteria [17], the maximum displacement from the normal hip-joint congruity was measured to grade the quality of fracture reduction and fixation: 0–1 mm displacement was regarded as anatomical, 2–3 mm as imperfect, and >3 mm as poor in plain film. All patients underwent a similar physical therapy program. As the patients' conditions permitted, early range of motion and non-weight-bearing exercises of the affected limb were conducted for 6 weeks, followed by partial weight-bearing exercises for 12 weeks. The rehabilitation protocol of full weight-bearing was individualized. After discharge, the patients were reviewed in the follow-up clinic at 2 months, 6 months, 1 year, and annually thereafter. The endpoint of the follow-up period was June 30, 2020.

### 2.2. Main Outcomes

The primary outcome was advanced secondary hip osteoarthritis requiring conversion to THA. We evaluated the clinical and radiologic findings, including crepitus, at the time of examination and conducted imaging studies for fragment displacement, joint incongruence, subluxation, and advanced posttraumatic arthritis to decide whether THA was required. At the final follow-up, which occurred after a minimum interval of 3 years after surgery, all files containing patients' information were updated. Candidate variable risk factors were selected after reviewing relevant literature about the postoperative outcomes of traumatic acetabular fractures. Categorical variables used were patient sex, body mass index (BMI), injury severity score (ISS), AO/OTA fracture and dislocation classification [16], weight-bearing dome involvement, type of central hip dislocation [18], marginal impaction, bladder injury, sciatic nerve injury, and quality of reduction. After preoperative CT scans were reviewed, weight-bearing dome involvement was classified into infratectal, juxtatectal, and transtectal fractures. Marginal impaction was defined as impaction of an osteochondral fragment into the subchondral bone. Median, first quartile, and third quartile were computed for continuous variables including age and the ISS.

### 2.3. Statistical Analysis

The chi-square test and Fisher's exact test were used to analyze the categorical variables between patients who underwent THA and those who did not. The Wilcoxon rank-sum test was used to analyze the continuous variables between survival to nonarthritis and advanced posttraumatic arthritis with conversion to THA. To plot the THA-free probability curve for the study subjects, we performed the Kaplan-Meier analysis. The hazard ratio (HR) of advanced stage posttraumatic arthritis and 95% confidence interval (CI) were assessed through Cox proportional hazard regression. SAS software version 9.4 (SAS Institute, Cary, NC, USA) was used for all statistical analyses. A *P* < 0.05 value was considered significant.

## 3. Results

### 3.1. Patient Characteristics

Fifty patients with acetabular fractures were included in this study. The mean follow-up period was 1337 days (range, 1101–1729 days). The baseline characteristics of the patients who completed the follow-up and the results of bivariate analysis are summarized in Table 1. The number of male patients was modestly higher than that of female patients (54% vs. 46%); patients' median age was 54.5 years; and the average ISS was 16. The proportion of patients with BMI < 24 and BMI ≥ 24 was 56% and 44%, respectively. According to the AO/OTA classification, about 60% of patients had transverse-type fractures (62B), and 40% had associated both column fractures (62C). The analysis of weight-bearing dome involvement revealed that most patients had transtectal fractures (62%), followed by juxtatectal (34%) and infratectal (4%) fractures. Initial CT or intraoperative Z-arthrotomy revealed an initial osteochondral injury of the femoral head in 24 patients (48%). During preoperative evaluation, bladder injury was identified in 7 patients (14%). Four patients (8%) had sciatic nerve injury.

### 3.2. Surgical Outcomes

According to Matta criteria, most patients had received anatomical reduction (22/50; 44%) or imperfect reduction (21/50; 42%). Reduction was poor in only 7/50 (14%). Patients who were defined as having an imperfect or poor reduction in X-ray underwent postoperative CT to assess the quality of reduction repeatedly. Therefore, there were 28 cases of repeated postoperative CT to qualify the reduction based on Matta criteria. At the final follow-up, we used the Majeed scoring system for functional evaluation; the score was excellent in 18 hips (36%), good in 20 (40%), fair in 11 (22%), and poor in 1 (2%). At the final follow-up, 11 patients (22%) had eventually undergone THA for advanced posttraumatic arthritis. Among these patients, male vs. female sex (81.8% vs. 46.2%), marginal impaction (81.8% vs. 38.5%), and sciatic nerve injury (27.3% vs. 2.56%) were significant risk factors.

**Figure 2:**
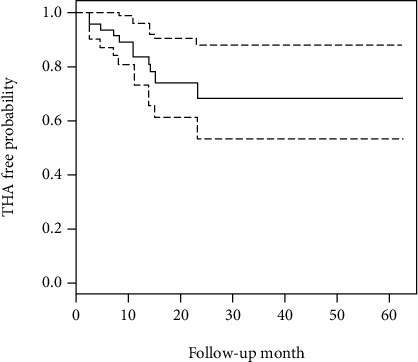
Advanced posttraumatic-arthritis-free probability in Kaplan-Meier analysis. The solid line is the probability, and the long-dotted line is the 95% confidence interval.

The diagnosis of end-stage secondary hip osteoarthritis was determined with multivariate Cox propensity hazard regression analysis (Table 2). According to the crude Cox proportional hazard regression, the risk factors were male sex (HR = 5.26, 95%CI = 1.13–5.26), marginal impaction (HR = 5.80, 95%CI = 1.23–27.3), and sciatic nerve injury (HR = 4.39, 95%CI = 1.15–16.8). Advanced posttraumatic arthritis was inversely related to age, BMI, ISS, AO/OTA fracture and dislocation classification, weight-bearing dome involvement, bladder injury, central hip dislocation type, and quality of reduction. In the adjusted Cox model, only male sex (HR = 6.00, 95%CI = 1.20–30.0) was significantly associated with traumatic arthritis after controlling for marginal impaction and sciatic nerve injury. The Kaplan-Meier survivorship analysis estimated that the cumulative probability of free-from-THA was 78% (95% CI, 73%–90%) at the 5-year follow-up (Figure 2).

## 4. Discussion

Although acetabular fractures with central hip dislocation have been described for decades, only a few studies have addressed the surgical results and the clinical outcome of this fracture. Unlike other acetabular fractures, central hip dislocations directly impact the acetabulum. In fact, these dislocations may involve more severe and underlying injuries within the hip joint than generally appreciated. Therefore, the goals of treatment for acetabular fractures with central hip dislocation are good long-term function and the avoidance of secondary hip osteoarthritis. In our study, the rate of secondary hip osteoarthritis after the surgical treatment of these fractures was 22% at 5 years. Male sex, sciatic nerve injury, and marginal impaction of the acetabular cartilage fragment were the risk factors most often associated with secondary hip osteoarthritis and THA; however, in the adjusted analysis, only male sex was significantly associated. At the final follow-up, the Majeed pelvic score was excellent in 18 hips (36%), good in 20 (40%), fair in 11 (22%), and poor in 1 (2%).

The acetabulum is formed by the convergence of the pubis, ilium, and ischium. Cartilage links these bones during infancy. After gradual ossification of the ossification center, the weight-bearing structure, including the anterior and posterior columns, is formed. The quadrilateral plate, which is the junction of the pubis, ilium, and ischium, is a non-weight-bearing structure and has lower bone density. Axial loading of the femur in abduction can lead to acetabular fractures with central hip dislocation. The intrapelvic impaction is usually transmitted from the femoral head to the acetabular columns and quadrilateral plate. Therefore, most central hip dislocations are accompanied by displacement of the quadrilateral plate fragment [14]. Several classifications of the fracture patterns and prognosis have been reported [15]. Intrapelvic displacement of the femoral head and the fracture pattern over the acetabular dome/quadrilateral plate have been mentioned, but these features do not predict outcome. Furthermore, none of them has been associated with the sequence of the surgical options or approach [15]. The AO/OTA fracture and dislocation classification was published in 2018 [16]. All our central hip dislocations were classified as AO/OTA 62B and 62C. This classification illustrates the acetabular fracture pattern and associated injury concisely and helps in deciding the surgical approach.

Surgical fixation of acetabular fractures with central hip dislocation and quadrilateral plate fragment is challenging. Judet et al. [4, 16] suggested operative approaches based on the fracture pattern. Because of the high complication rate associated with extensile approaches [19], the ilioinguinal approach has become the most widely used intrapelvic approach for management of the quadrilateral plate fragment. Letournel [16] introduced the ilioinguinal approach to access the medial wall of the acetabulum. The clinical outcomes of the ilioinguinal approach were superior to those of the extensile approaches; however, this approach was associated with postoperative complications such as nerve palsy, laceration of the femoral artery, and disruption of the inguinal ligament [19, 20], so alternative approaches have been introduced recently. The modified Stoppa approach has lower complication rates and better functional outcomes than does the ilioinguinal approach. Due to the difficulty in treating posterior column displacement with the modified Stoppa approach, the use of dual approaches has been suggested [21, 22]. To access the posterior column and the posterior wall, the KL approach with or without trochanteric osteotomy, which achieves rigid fixation and is associated with a low complication rate, is recommended [23, 24]. In our study, we combined the modified Stoppa approach and the KL approach to reduce joint congruity when accessing the posterior column or posterior wall. According to Matta criteria, anatomical reduction was achieved in 44% of the patients, and reduction was poor in only 14%. Also, the dual approach provided adequate surgical fields and high-quality reduction.

The outcomes of surgical treatment of acetabular fractures have been reported since the 1960s. Judet et al. [4] reported an 18%–24% rate of secondary hip osteoarthritis after surgical treatment of acetabular fractures. Two large retrospective studies have reported 21% and 18% rates of secondary hip osteoarthritis at 20-year follow-up after surgical treatment [25, 26]. Recently, a registry study in Germany reported a 19.8% rate of secondary hip osteoarthritis after acetabular fractures [27]. However, all the studies have reported only the outcomes and predictors for the subtypes of acetabular fractures; no comprehensive study with long-term follow-up of results and risk factors of secondary hip osteoarthritis after surgical treatment of acetabular fractures with central hip dislocation has been reported. In our study, at a follow-up of as long as 5 years, 22% of patients had advanced posttraumatic arthritis, an outcome like that in other studies [25, 26].

In the studies of all subtypes of acetabular fractures, the predictors for advanced posttraumatic arthritis varied. In some cases, the medial-wall fragment at the pelvis associated with femoral head impaction was rotated and impacted against the underlying cancellous bone along the fracture margin [28]. Marginal impaction creates significant joint incongruity and cartilage damage if not identified and addressed during fixation of the acetabular fracture and can lead to early arthritis [29]. Tannast et al. [25] reported significant associations of secondary osteoarthritis with patients age > 40 years, postoperative incongruence of the acetabular roof, involvement of the posterior acetabular wall, acetabular impaction, a femoral head cartilage lesion, initial displacement of the articular surface of >20 mm, and use of the extended iliofemoral approach. On the other hand, injury to the femoral head and acetabular impaction were the strongest predictors of long-term survival of the native hip joint after acetabular fractures [26]. In a multicenter registry study, the likelihood of THA was higher in cases of posterior wall involvement and femoral head impaction [27]. By contrast, acetabular fractures with central hip dislocation may create severe impaction injuries to the cartilage on both surfaces of the hip joint. Our study demonstrates that one of the independent noncontrollable factors associated with secondary hip osteoarthritis is marginal impaction on the acetabulum.

According to multiple regression analyses, preoperative sciatic nerve injury and male sex are also independent negative predictors of conversion to THA. The proportion of patients having acetabular fractures with nerve injuries diagnosed preoperatively was 4% [30]; the injuries may result from blunt contusion to the sciatic nerve, laceration, or stretching of the nerve over the dislocated femoral head [31]. Our study also found a higher prevalence of preoperative sciatic nerve injury; perhaps, the higher axial loading from the femoral head to the acetabulum not only can induce central hip dislocation but can also stretch the sciatic nerve root. This phenomenon might explain why multiple regression analyses identified preoperative sciatic nerve injury as a risk factor for secondary osteoarthritis. Male gender also was a risk factor of end-stage traumatic osteoarthritis. A possible explanation for this finding is that more males than females had an occupation requiring heavy manual labor; heavy lifting is reportedly associated with an increased risk of hip osteoarthritis, and it might increase the incidence of joint failure [32]. We did not routinely set a limit on types of patients' occupational activity and timing of return to work. Longer follow-ups are required to confirm the identified risk factors. In our study, marginal impaction, preoperative sciatic injury, and male sex were critical risk factors of secondary hip osteoarthritis. This finding indicated the importance of articular cartilage injury and preoperative condition rather than reduction of fracture gap at postoperative follow-up. Therefore, we recommend precise examination of sciatic nerve function and a CT scan for evaluating the presence of marginal impaction.

## 5. Limitations

The present study has limitations: first, it had a retrospective design and was based on a single surgeon's experience. Second, the incidence of acetabular fractures with central hip dislocation is lower than that of other fractures, so the number of patients was few. Third, arthritic changes progress slowly to cause deterioration in clinical performance. As we set a minimum of only 3-year follow-up in our study, the conclusion regarding the predictors of advanced posttraumatic arthritis with a need for THA was not definitive. Fourth, the quality of reduction was assessed according to pelvic radiographs and graded based on Matta criteria in this study. Verbeek et al. [34] have indicated that CT is superior to plain film in detecting residual fracture displacement and is more predictive for hip survivorship. In our study, postoperative CT was only performed in cases with imperfect and poor reduction quality to more accurately evaluate the progressive hip arthritis and assess the extent of fracture displacement. Therefore, only 28 patients underwent postoperative CT. Despite these limitations, our study has methodological strengths: it helps fill the void of studies addressing the outcomes of patients with acetabular fractures with central hip dislocation; most of the relevant literature consists of case series or case reports. The main strength of this study is that it presents the clinical outcome and negative predictors of conversion to THA of the largest case series of acetabular fractures with central hip dislocation.

## 6. Conclusions

We have reported a consecutive case series of acetabular fractures with central hip dislocation and surgical fixation to evaluate the risk factors of secondary hip osteoarthritis requiring total hip arthroplasty. Marginal impaction was found a predictor of advanced posttraumatic arthritis. Preoperative sciatic nerve injury and male sex were found risk factors, probably because of the high axial loading mechanism and occupation requiring heavy manual labor and lifting. In selected patients, the presence of such risk factors can guide treatment toward earlier conversion to total hip arthroplasty.

## Figures and Tables

**Table 1:** Distribution of age, sex, and clinical report in study subjects.

Patient characteristics	Total number *N* = 50 (%)	Survival to THA *N* = 39 (%)	Conversion to THA *N* = 11 (%)	*P* value^1^
Sex				0.046^∗^
Female	23 (46.0%)	21 (53.9%)	2 (18.2%)	
Male	27 (54.0%)	18 (46.2%)	9 (81.8%)	
BMI				0.91
<24	28 (56.0%)	22 (56.4%)	6 (54.5%)	
≥24	22 (44.0%)	17 (43.6%)	5 (45.5%)	
AO/OTA classification				0.31
62B	30 (60.0%)	25 (64.1%)	5 (45.5%)	
62C	20 (40.0%)	14 (35.9%)	6 (54.5%)	
Infra/juxta/transtectal				0.57
1	2 (4.0%)	1 (2.56%)	1 (9.09%)	
2	17 (34.0%)	14 (35.9%)	3 (27.3%)	
3	31 (62.0%)	24 (61.5%)	7 (63.6%)	
Marginal impaction				0.02^∗^
No	26 (52.0%)	24 (61.5%)	2 (18.2%)	
Yes	24 (48.0%)	15 (38.5%)	9 (81.8%)	
Bladder injury				0.17
No	43 (86.0%)	34 (89.7%)	8 (72.7%)	
Yes	7 (14.0%)	5 (10.3%)	3 (27.3%)	
Sciatic nerve injury				0.03^∗^
No	46 (92.0%)	38 (97.4%)	8 (72.7%)	
Yes	4 (8.0%)	1 (2.56%)	3 (27.3%)	
Central dislocation type				0.15
1	15 (30.0%)	14 (35.9%)	1 (9.09%)	
2	22 (44.0%)	17 (43.6%)	5 (45.5%)	
3	13 (26.0%)	8 (20.5%)	5 (45.5%)	
Matta reduction criteria				0.055
Anatomical	22 (44.0%)	19 (48.7%)	3 (27.3%)	
Imperfect	21 (42.0%)	17 (43.6%)	4 (36.4%)	
Poor	7 (14.0%)	3 (7.69%)	4 (36.4%)	
Age, y, mean (Q1, Q3)	54.5 (37, 66)	52.5 (37, 66)	55 (32, 72)	0.90^2^
ISS, mean (Q1, Q3)	16 (10, 20)	16 (10, 20)	18 (16, 20)	0.52^2^

^1^Chi-square test/Fisher's exact test; ^2^Wilcoxon rank-sum test.

**Table 2:** Advanced stage posttraumatic arthritis-associated risk factor using Cox proportional hazard regression.

	Crude HR (95% CI)	Adjusted HR (95% CI)
Age		
<65	Ref.	
65+	1.49 (0.44-5.09)	
Sex		
Female	Ref.	Ref.
Male	5.26 (5.26-1.13)^∗^	6.00 (1.20-30.0)^∗^
BMI		
<24	Ref.	
≥24	0.97 (0.30-3.19)	
ISS		
<17	Ref.	
17+	1.64 (0.48-5.62)	
AO/OTA classification		
62B	Ref.	
62C	1.94 (0.59-6.38)	
Infra/juxta/transtectal		
1	3.15 (0.32-30.7)	
2	Ref.	
3	1.50 (0.39-5.80)	
Marginal impaction		
No	Ref.	Ref.
Yes	5.80 (1.23-27.3)^∗^	3.91 (0.77-19.8)
Bladder injury		
No	Ref.	
Yes	2.67 (0.70-10.1)	
Sciatic nerve injury		
No	Ref.	Ref.
Yes	4.39 (1.15-16.8)^∗^	3.68 (0.84-16.1)
Central dislocation type		
1	Ref.	
2	2.68 (0.31-22.9)	
3	4.74 (0.55-41.0)	
Matta reduction criteria		
Anatomical	Ref.	
Imperfect	1.18 (0.26-5.31)	
Poor	3.68 (0.82-16.5)	

^∗^*P* < 0.05, ^∗∗^*P* < 0.01.

## Data Availability

The data used to support the findings of this study are included within the article.

## Abbreviations

THA: Total hip arthroplasty
AO/OTA: Arbeitsgemeinschaft für Osteosynthesefragen/Orthopaedic Trauma Association
KL: Kocher-Langenbeck
CT: Computed tomography
BMI: Body mass index
ISS: Injury severity score
HR: Hazard ratio
CI: Confidence interval.
